# Clopidogrel and Aspirin Initiated Between 24 to 72 Hours for Mild Ischemic Stroke: A Subgroup Analysis of the INSPIRES Randomized Clinical Trial

**DOI:** 10.1001/jamanetworkopen.2024.31938

**Published:** 2024-09-06

**Authors:** Yuetong Liu, Jinguo Zhao, Ying Gao, Weiqi Chen, S. Claiborne Johnston, Philip M. Bath, Pierre Amarenco, Hongyi Yan, Xuan Wang, Yingying Yang, Tingting Wang, Yongjun Wang, Yuesong Pan, Yilong Wang

**Affiliations:** 1 Department of Neurology, Beijing Tiantan Hospital, Capital Medical University, Beijing, China; 2 Department of Neurology, Weihai Wendeng District People’s Hospital, Shandong, China; 3 Department of Neurology, University of California, San Francisco; 4 Stroke Trials Unit, Division of Mental Health and Clinical Neuroscience, University of Nottingham, Nottingham, United Kingdom; 5 Department of Neurology and Stroke Center, Bichat Hospital, Assistance Publique–Hôpitaux de Paris, University of Paris, Paris, France; 6 Population Health Research Institute, McMaster University, Hamilton, Ontario, Canada; 7 China National Clinical Research Center for Neurological Diseases, Beijing Tiantan Hospital, Capital Medican University, Beijing, China; 8 Advanced Innovation Center for Human Brain Protection, Capital Medical University, Beijing, China; 9 National Center for Neurological Disorders, Shanghai, China; 10 Research Unit of Artificial Intelligence in Cerebrovascular Disease, Chinese Academy of Medical Sciences, Beijing, China; 11 Beijing Laboratory of Oral Health, Capital Medical University, Beijing, China; 12 Chinese Institute for Brain Research, Beijing, China

## Abstract

**Importance:**

Prior trials showed that dual antiplatelet therapy could reduce the risk of early new stroke in patients with acute mild ischemic stroke or transient ischemic attack (TIA) within 24 hours of symptom onset. However, it is currently uncertain whether dual antiplatelet therapy can reduce the risk of early new stroke in patients with a more delayed initiation time window.

**Objective:**

To evaluate the efficacy and safety of clopidogrel and aspirin among patients with mild ischemic stroke or TIA when initiated within 24 hours, from more than 24 hours to 48 hours, and from more than 48 hours to 72 hours.

**Design, Setting, and Participants:**

The Intensive Statin and Antiplatelet Therapy for Acute High-Risk Intracranial or Extracranial Atherosclerosis randomized clinical trial was a double-blind, placebo-controlled, multicenter, 2-by-2 factorial randomized clinical trial conducted at 222 hospitals in China from September 17, 2018, to October 15, 2022. All patients with acute mild ischemic stroke and TIA were included in this subgroup analysis and categorized into 3 groups according to time from symptom onset to randomization (group 1: ≤24 hours; group 2: >24 to ≤48 hours; and group 3: >48 to 72 hours). Patients were followed up for 90 days.

**Interventions:**

All patients received clopidogrel combined with aspirin (clopidogrel 300 mg loading dose on day 1, followed by 75 mg daily on days 2 to 90, and aspirin 100 to 300 mg on the first day, 100 mg daily for days 2 to 21, and then a matching aspirin placebo for days 22 to 90) or aspirin alone (100 to 300 mg on day 1 and then 100 mg daily for days 2 to 90) within 72 hours after symptom onset.

**Main Outcomes and Measures:**

The primary outcome was new stroke (ischemic or hemorrhagic) within 90 days. The primary safety outcome was moderate-to-severe bleeding, according to Global Utilization of Streptokinase and Tissue Plasminogen Activator for Occluded Coronary Arteries criteria.

**Results:**

This analysis included a total of 6100 patients (3050 in the clopidogrel-aspirin group and 3050 in the aspirin group). The median age was 65 years (IQR, 57-71 years), and 3915 patients (64.2%) were male. In the population with time to randomization of 24 hours or less, stroke occurred in the next 90 days in 97 of 783 patients (12.4%); among those randomized from more than 24 hours to 48 hours, in 211 of 2552 patients (8.3%); and among those randomized from more than 48 hours to 72 hours, in 193 of 2765 patients (7.0%). The clopidogrel-aspirin group had a lower risk of new stroke within 90 days compared with the aspirin alone group both in patients with time to randomization of from 48 to 72 hours (5.8% vs 8.2%; hazard ratio [HR], 0.70 [95% CI, 0.53-0.94]), of more than 24 to 48 hours (7.6% vs 8.9%; HR, 0.85 [95% CI, 0.65-1.12]), and of 24 hours or less (11.5% vs 13.4%; HR, 0.83 [95% CI, 0.55-1.25]) (*P* = .38 for interaction). Among those with time to randomization of more than 48 to 72 hours, moderate-to-severe bleeding occurred in 12 patients (0.9%) in the clopidogrel-aspirin group and in 6 patients (0.4%) in the aspirin-alone group (HR, 2.00 [95% CI, 0.73-5.43]), while moderate-to-severe bleeding in those with time to randomization of more than 24 hours to 48 hours occurred in 9 patients (0.7%) in the clopidogrel-aspirin group and in 4 patients (0.3%) in the aspirin-alone group (HR, 2.25 [95% CI, 0.68-7.39]) and in those with time to randomization of within 24 hours, occurred in 6 patients (1.5%) in the clopidogrel-aspirin group and in 3 patients (0.8%) in the aspirin-alone group (HR, 1.57 [95% CI, 0.36-6.83]) (*P* = .92 for interaction).

**Conclusions and Relevance:**

In this randomized clinical trial of antiplatelet therapy in China, patients with mild ischemic stroke or TIA had consistent benefit from dual antiplatelet therapy with clopidogrel and aspirin vs aspirin alone when initiated within 72 hours after symptom onset, with a similar increase in the risk of moderate-to-severe bleeding. Patients should receive dual antiplatelet therapy with clopidogrel and aspirin within 72 hours after symptom onset.

**Trial Registration:**

ClinicalTrials.gov Identifier: NCT03635749

## Key Points

**Question:**

Is dual antiplatelet therapy with clopidogrel and aspirin effective when initiated up to 72 hours after mild ischemic stroke and transient ischemic attack (TIA)?

**Findings:**

In this subgroup analysis of a randomized clinical trial including 6100 patients with mild ischemic stroke or TIA, treatment with clopidogrel and aspirin showed similar efficacy and safety compared with aspirin alone among patients with mild ischemic stroke or TIA when initiated from less than 24 hours to 72 hours after symptom onset. A similar increase in risk of moderate-to-severe bleeding was shown.

**Meaning:**

The results of this study suggest that patients with mild ischemic stroke or TIA can benefit from dual antiplatelet therapy with clopidogrel and aspirin when initiated up to 72 hours after symptom onset, without an excess of risk of moderate-to-severe bleeding.

## Teaser

This randomized clinical trial evaluates the efficacy and safety of dual antiplatelet therapy with clopidogrel and aspirin compared with aspirin alone among patients in China with mild ischemic stroke or transient ischemic attack when initiated up to 72 hours after symptom onset.

## Introduction

Acute mild ischemic stroke or transient ischemic attack (TIA) is associated with a high risk of subsequent stroke within 90 days (in approximately 8% to 10% of patients).^1,2,3^ Currently, dual antiplatelet therapy (DAPT) is considered to be a key strategy to prevent early new stroke for these patients.^4^ The efficacy and safety of DAPT with clopidogrel and aspirin in reducing the risk of early new stroke have been evaluated in patients with acute minor ischemic stroke or high-risk TIA within 24 hours after symptom onset.^5,6,7^ Based on these trials, the current guidelines recommend early (within 24 hours) and short (21 or 30 days) combination treatment with clopidogrel and aspirin for patients with minor stroke or TIA.^8,9^ These findings provided a feasible but narrow time window for secondary stroke prevention through DAPT. However, there is uncertainty about the benefits and risks of DAPT for these patients with a prolonged initiation time window.

In the Intensive Statin and Antiplatelet Therapy for Acute High-Risk Intracranial or Extracranial Atherosclerosis (INSPIRES) trial, DAPT with clopidogrel and aspirin initiated within 72 hours was superior to aspirin alone with a lower risk of new stroke within 90 days but a higher risk of moderate-to-severe bleeding.^10^ The INSPIRES trial included 2552 patients with time to randomization of more than 24 hours to 48 hours and 2737 patients randomized to more than 48 hours to 72 hours after symptom onset. For patients with acute mild stroke or TIA, the risk of new stroke was most pronounced in the first day of ictus and remained high within the subsequent 24 to 72 hours,^5,11,12^ but a lower bleeding risk may have offset the reduced benefit from delayed initiation of DAPT.^13^ However, to our knowledge, there are few data on efficacy and safety of DAPT over various initiation time windows, and currently, no large-scale clinical trial has evaluated the effect of DAPT focused on patients within 24 to 72 hours after symptom onset. In this subgroup analysis, we aimed to explore the efficacy and safety of DAPT with clopidogrel and aspirin in reducing the risk of new stroke at 90 days in patients with mild ischemic stroke and high-risk TIA among different initiation time windows of DAPT, especially when initiated from more than 24 hours to 48 hours and from more than 48 hours to 72 hours.

## Methods

### Study Design

The data were derived from the INSPIRES trial. The rationale, design, and methods of this trial have been previously described in detail.^14^ The protocol and statistical analysis plan are presented in Supplement 1. The INSPIRES trial was a double-blind, placebo-controlled, multicenter, and 2 × 2 factorial randomized clinical trial, conducted at 222 centers in China from September 17, 2018, to October 15, 2022. The 2 factors for this 2 × 2 factorial design were antiplatelet therapy and statin therapy. The objective of INSPIRES was to assess whether DAPT with clopidogrel and aspirin vs aspirin alone and immediate intensive statin therapy vs 3-day delayed intensive statin therapy could reduce the risk of new stroke within 90 days in acute mild ischemic stroke or high-risk TIA of atherosclerotic origin within 72 hours after symptom onset. The INSPIRES trial was approved by the ethics committee at each study center. All participants or their legal proxies provided written informed consent before enrollment. The study followed the Consolidated Standards of Reporting Trials (CONSORT) reporting guideline.

In the INSPIRES trial, eligible patients were aged 35 to 80 years, had either an acute mild ischemic stroke (National Institutes of Health Stroke Scale [NIHSS] score ≤5; range, 0 to 42, with higher scores indicating more severe stroke) or a high-risk TIA (age, blood pressure, clinical features, duration of symptoms, and diabetes score ≥4; range, 0 to 7, with higher scores indicating greater risk of stroke) between 24 and 72 hours after onset or had an ischemic stroke (NIHSS score of 4 to 5) within 24 hours of symptom onset. Acute ischemic stroke and TIA were diagnosed according to the American Heart Association and American Stroke Association criteria^15^ and confirmed by magnetic resonance imaging or brain computed tomography. Ischemic stroke was diagnosed if a patient had an episode of neurological dysfunction caused by focal cerebral, spinal, or retinal infarction. Transient ischemic attack was diagnosed if a patient had focal arterial ischemia with transient symptoms (lasting <24 hours) and without evidence of infarction by pathology or imaging. Patients were not eligible if they received intravenous thrombolytic therapy or mechanical thrombectomy or defibrillation, anticoagulation, or antiplatelet therapy other than clopidogrel or aspirin. Additional exclusion criteria included clear cardiogenic ischemic cerebrovascular disease, nonvascular intracranial disease, previous history of intracranial hemorrhage, use of DAPT within 14 days prior to randomization, or severe liver or renal dysfunction prior to randomization. More detailed inclusion and exclusion criteria are provided in the protocol.^14^ Patients were randomly assigned to receive clopidogrel combined with aspirin (clopidogrel 300 mg loading dose on day 1, followed by 75 mg daily on days 2 to 90, and aspirin 100 to 300 mg on the first day, 100 mg daily for days 2 to 21, and then a matching aspirin placebo for days 22 to 90) or clopidogrel placebo combined with aspirin (100 to 300 mg on day 1 and then 100 mg daily for days 2 to 90) in a 1:1 ratio within 72 hours of symptom onset. Patients received the initial doses as soon as feasible within 1 hour after being assigned to a trial group.

There was no interaction between antiplatelet and statin treatments, and the results of efficacy and safety of DAPT with clopidogrel and aspirin compared with aspirin alone were presented previously.^10^ An exploratory analysis of data to explore the effect of DAPT in patients with different times from symptom onset to randomization was performed and reported in this study. The present analysis was performed to compare patients with times from symptom onset to randomization of 24 hours or less, more than 24 hours to 48 hours, and more than 48 hours to 72 hours. Symptom onset of a qualifying event was defined as the point at which the patient reported no longer being in a normal condition.

### Outcome

The primary efficacy outcome was any new stroke within 90 days, including hemorrhagic and ischemic stroke, as in the parent INSPIRES trial. Secondary efficacy outcomes included composite vascular events (stroke, myocardial infarction, and cardiovascular death), ischemic stroke, myocardial infarction, vascular death, and poor functional outcome (modified Rankin scale score of 2 to 6; range, 0 to 6, with higher scores indicating more disability and a score of 6 indicating death). The primary safety outcome was moderate-to-severe bleeding defined by criteria from the Global Utilization of Streptokinase and Tissue Plasminogen Activator for Occluded Coronary Arteries trial within 90 days.^16^ Secondary safety outcomes included intracranial hemorrhage, any bleeding, and all cause death. All efficacy and safety analyses were based on independent clinical-event adjudication committee-assessed events.

### Statistical Analysis

This post hoc analysis was exploratory and hypothesis generating across prespecified subgroups. Baseline characteristics were presented by patients with different times to randomization (≤24 hours, >24 to ≤48 hours, and >48 to 72 hours) and antiplatelet therapy groups (DAPT vs aspirin alone). Continuous variables were presented as medians with IQRs and compared using the Kruskal-Wallis test. Categorical variables were presented as percentages and compared using the χ^2^ test.

All efficacy and safety outcomes were conducted based on the intention-to-treat principle and included all randomized patients. Outcomes were compared among time-to-randomization groups and between antiplatelet therapy groups across different time-to-randomization groups. We used the Cox proportional hazards regression model to calculate hazard ratios (HRs) and 95% CIs for stroke, combined vascular events, ischemic stroke, myocardial infarction, vascular death, moderate-to-severe bleeding, any bleeding, intracranial hemorrhage, and all-cause mortality outcomes. The Cox proportional hazards regression model assumption was affirmed by including a time-dependent covariate with the interaction of subgroups and a logarithmic function of survival time in the model. The Kaplan-Meier method was used to evaluate the survival curve of stroke. For the outcome of poor functional outcome, log-normal regression was used to calculate relative risk (RR) and 95% CI. For each outcome, 2 models were performed: first, an unadjusted model and second, adjusted with covariates comprising age, sex, medical history (previous ischemic stroke, diabetes, hypertension, or dyslipidemia), current smoking, systolic and diastolic blood pressure at admission, use of antiplatelet and lipid-lowering agents before qualifying event, qualifying event, symptomatic artery stenosis, NIHSS score, and statin treatment assignment. To exclude the potential influence of immediate or delayed statin therapy due to the 2 × 2 factorial design of the trial, statin treatment assignment was adjusted in the model. The interaction between the time-to-randomization group and antiplatelet treatment assignment on all outcomes was evaluated by including terms for treatment assignment (clopidogrel-aspirin group or aspirin-alone group), time-to-randomization group (≤24 hours, >24 to ≤48 hours, and >48 to 72 hours), and treatment × time-to-randomization group interaction as covariates in the Cox proportional hazards or log-normal regression models. We also evaluated the influence of time to randomization as a continuous variable on the effect of clopidogrel with aspirin vs aspirin alone for the primary outcome of new stroke, assuming a linear relationship. Missing data for patients lost to follow-up at 90 days were treated as censored for outcomes of vascular events and mortality, while complete case data were used for poor functional outcome.

Among primary events in the time-to-randomization groups, the power was 10% (HR, 0.87) in the group of 24 hours or less, 27% (HR, 0.83) for 24 hours to 48 hours, and 66% (HR, 0.71) for more than 48 hours to 72 hours to detect an effect. No adjustment for multiple comparisons was made, and all *P* values were nominal, since all analyses presented were exploratory, using 2-sided *P* < .05 as the threshold for statistical significance. All statistical analyses were conducted using SAS, version 9.4 (SAS Institute Inc).

## Results

### Participants

The INSPIRES trial included a total of 6100 patients (n = 3050 in the clopidogrel-aspirin group and n = 3050 in the aspirin group) with acute mild ischemic stroke and high-risk TIA. The median age was 65 years (IQR, 57-71 years), 2185 patients (35.8%) were female, and 3915 (64.2%) were male. Among the patients, 783 (12.8%) were with time to randomization of 24 hours or less (268 female [34.2%]; 515 male [65.8%]), 2552 (41.8%) of more than 24 hours to 48 hours (906 female [35.5%]; 1646 male [64.5%]), and 2765 patients (45.3%) of more than 48 hours to 72 hours (1011 female [36.6%]; 1754 male [63.4%]) (eFigure in Supplement 2). A total of 4 patients in the clopidogrel-aspirin group and 2 in the aspirin group were lost to follow-up at 90 days. Seven patients had missing data regarding functional outcome.

The baseline characteristics of patients in different time-to-randomization groups are shown in eTable 1 in Supplement 2. Patients with a longer time from onset to randomization had higher proportions of history of hypertension and use of antihypertensive agents, and a lower proportion were current smokers. Among patients with time to randomization of more than 24 hours to 48 hours and more than 48 hours to 72 hours, patients were more likely to have acute multiple infarctions, with a higher proportion of the NIHSS score of 3 or less as compared with those with time to randomization of 24 hours or less. Baseline characteristics were well balanced between the 2 antiplatelet therapy groups across different time-to-randomization groups (Table 1).

**Table 1.** Baseline Characteristics of Patients by the Time-to-Randomization Group

Characteristic	Time to randomization, h					
	≤24		>24 to ≤48		>48 to 72	
	Clopidogrel with aspirin (n = 401)	Aspirin (n = 382)	Clopidogrel with aspirin (n = 1255)	Aspirin (n = 1297)	Clopidogrel with aspirin (n = 1394)	Aspirin (n = 1371)
Age, median (IQR), y	65 (57-71)	65 (56-71)	64 (56-71)	66 (57-72)	65 (57-71)	65 (57-71)
Sex, No. (%)						
Female	139 (34.7)	129 (33.8)	434 (34.6)	472 (36.4)	490 (35.2)	521 (38.0)
Male	262 (65.3)	253 (66.2)	821 (65.4)	825 (63.6)	904 (64.8)	850 (62.0)
Blood pressure, median (IQR), mmHg						
Systolic	148 (135-160)	150 (135-165)	146 (132-160)	146 (132-160)	145 (132-160)	146 (133-160)
Diastolic	86 (80-94)	88 (80-96)	85 (80-94)	85 (78-94)	84 (77-92)	85 (78-93)
Medical history, No. (%)						
Hypertension	252 (62.8)	255 (66.8)	831 (66.2)	847 (65.3)	964 (69.2)	934 (68.1)
Diabetes	107 (26.7)	89 (23.3)	349 (27.8)	366 (28.2)	374 (26.8)	373 (27.2)
Dyslipidemia	18 (4.5)	17 (4.5)	46 (3.7)	48 (3.7)	39 (2.8)	58 (4.2)
Previous ischemic stroke	120 (29.9)	116 (30.4)	368 (29.3)	377 (29.1)	413 (29.6)	415 (30.3)
Current smoking, No. (%)	123 (30.7)	131 (34.3)	395 (31.5)	372 (28.7)	374 (26.8)	388 (28.3)
Use of agents before qualifying event, No. (%)						
Aspirin	62 (15.5)	52 (13.6)	158 (12.6)	180 (13.9)	170 (12.2)	171 (12.5)
Clopidogrel	1 (0.2)	7 (1.8)	9 (0.7)	5 (0.4)	11 (0.8)	10 (0.7)
Lipid lowering	47 (11.7)	40 (10.5)	115 (9.2)	127 (9.8)	134 (9.6)	124 (9.0)
Qualifying event, No. (%)						
TIA	74 (18.5)	68 (17.8)	161 (12.8)	190 (14.7)	164 (11.8)	144 (10.5)
Acute single infarction	79 (19.7)	69 (18.1)	243 (19.4)	246 (19.0)	266 (19.1)	271 (19.8)
Acute multiple infarctions	248 (61.8)	245 (64.1)	851 (67.8)	861 (66.4)	964 (69.2)	956 (69.7)
Symptomatic stenosis ≥50%, No. (%)						
Yes	315 (82.2)	324 (86.4)	1010 (82.0)	1042 (81.9)	1123 (82.0)	1101 (82.5)
No	68 (17.8)	51 (13.6)	222 (18.0)	231 (18.1)	247 (18.0)	234 (17.5)
NIHSS score in qualifying ischemic stroke, No. (%)^a^						
≤3	208 (63.6)	213 (67.8)	852 (77.9)	845 (76.3)	947 (77.0)	968 (78.9)
>3	119 (36.4)	101 (32.2)	242 (22.1)	262 (23.7)	283 (23.0)	259 (21.1)
ABCD2 score in qualifying TIA, No. (%)^b^						
4 or 5	58 (78.4)	59 (86.8)	129 (80.1)	140 (73.7)	139 (84.8)	116 (80.6)
>5	16 (21.6)	9 (13.2)	32 (19.9)	50 (26.3)	25 (15.2)	28 (19.4)
Statin therapy, No. (%)						
Immediate intensive statin	207 (51.6)	184 (48.2)	634 (50.5)	655 (50.5)	684 (49.1)	686 (50.0)
Delayed intensive statin	194 (48.4)	198 (51.8)	621 (49.5)	642 (49.5)	710 (50.9)	685 (50.0)

Abbreviations: ABCD2, age, blood pressure, clinical features, duration of symptoms, and diabetes; NIHSS, National Institutes of Health Stroke Scale; TIA, transient ischemic attack.

^a^ Scores range from 0 to 42 for patients with ischemic stroke, with higher scores indicating more severe stroke.

^b^ Scores range from 0 to 7, with higher scores indicating greater risk of stroke.

### Efficacy Outcomes

**Figure 1.**
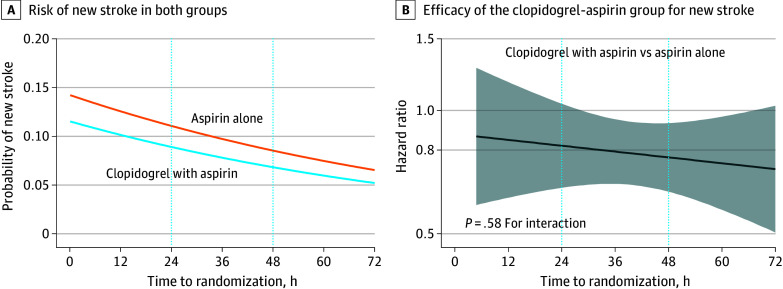
Risk of New Stroke (Primary Efficacy Outcome) and Efficacy of Clopidogrel With Aspirin for New Stroke by Time to Randomization The shaded area indicates 95% CIs of the effect of clopidogrel and aspirin by time to randomization.

**Figure 2.**
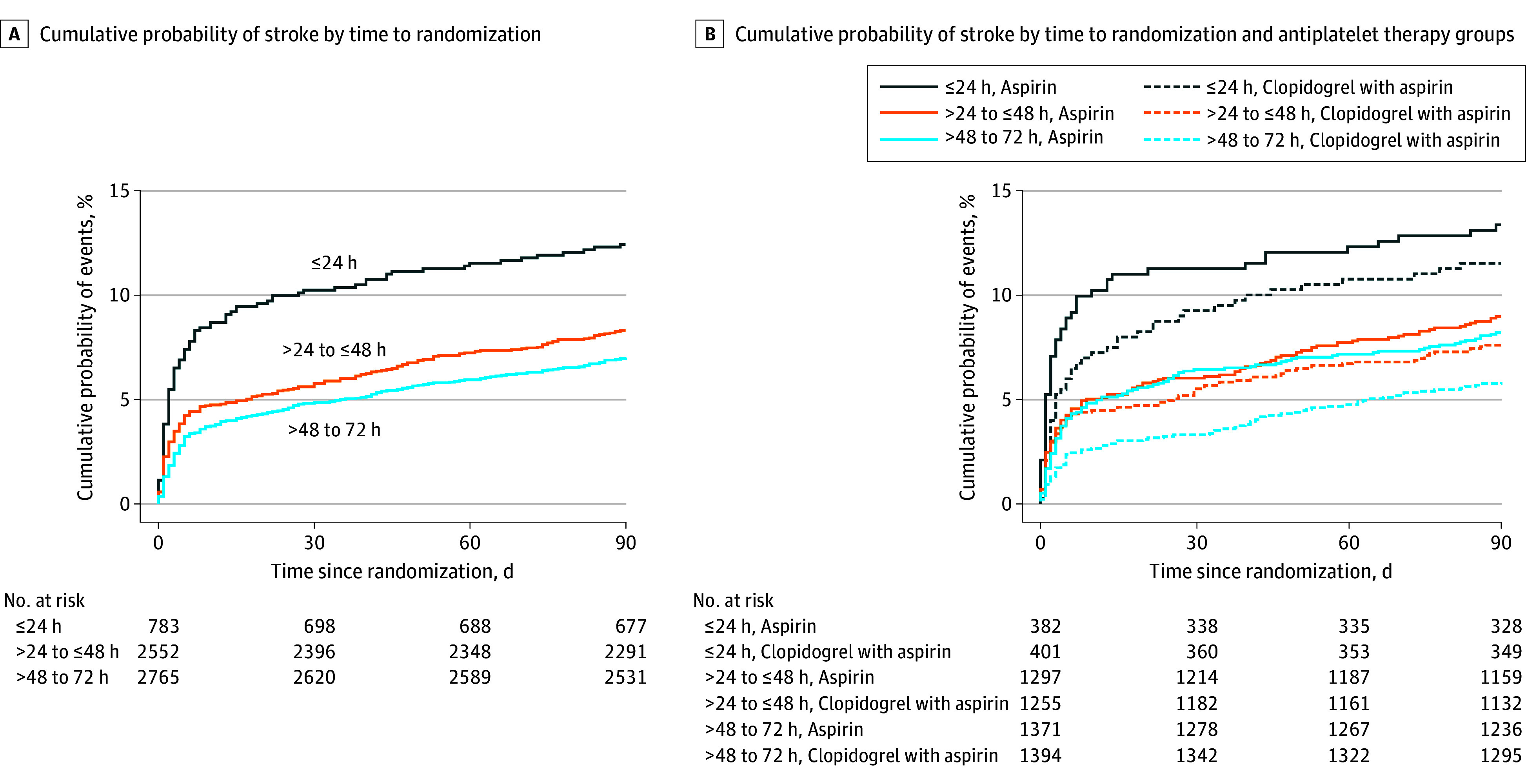
Kaplan-Meier Survival Curves of Cumulative Probability of Stroke (Primary Efficacy Outcome) by Time to Randomization and Antiplatelet Therapy Groups

In the population with time to randomization of 24 hours or less, new stroke occurred within 90 days in 97 patients (12.4%), in 211 patients (8.3%) in the group of more than 24 hours to 48 hours, and in 193 patients (7.0%) in the group of more than 48 hours to 72 hours. Compared with time to randomization of 24 hours or less, time to randomization of more than 24 hours to 48 hours (HR, 0.64 [95% CI, 0.50-0.81]; *P* < .001) and of more than 48 to 72 hours (HR, 0.52 [95% CI, 0.41-0.67]; *P* < .001) was associated with lower risk of new stroke within 90 days after adjusting for potential confounding factors (eTable 2 in Supplement 2 and Figure 1A). Figure 2A shows the Kaplan-Meier survival curve of new stroke as the primary end point by different times to randomization.

For the primary efficacy outcome, similar relative efficacy of the clopidogrel-aspirin group was observed across the time-to-randomization groups (Figure 1B). No significant interaction between antiplatelet treatment assignment and time-to-randomization group for the primary efficacy outcome of new stroke was identified (Table 2), indicating consistent efficacy of clopidogrel with aspirin vs aspirin alone among patients with different times to randomization. In patients with time to randomization of more than 48 to 72 hours, the clopidogrel-aspirin group had a lower risk of new stroke within 90 days compared with the aspirin-alone group (5.8% vs 8.2%; HR, 0.70 [95% CI, 0.53-0.94]; risk difference, −1.77% [95% CI, −1.83% to −1.70%]), with a number needed to treat of 56 to avoid 1 new stroke at day 90 (Table 2 and Figure 2). The effect size was numerically larger than that in patients with times to randomization of more than 24 to 48 hours (7.6% vs 8.9%; HR, 0.85 [95% CI, 0.65-1.12]; risk difference, −1.19% [95% CI, −1.24% to −0.99%]; number needed to treat, 84) and of 24 hours or less (11.5% vs 13.4%; HR, 0.83 [95% CI, 0.55-1.25]; risk difference, −1.37% [95% CI, −2.35% to 16.22%]; number needed to treat, 73) (*P* = .38 for interaction). This indicates that the risk of new stroke may be reduced with clopidogrel and aspirin by 15% when initiated within more than 24 hours to 48 hours and by 30% when initiated within more than 48 hours to 72 hours after symptom onset. Similar results were observed for the secondary efficacy outcomes of composite cardiovascular event and ischemic stroke. The functional outcome data at 90 days were missing in 2 patients in the group of 24 hours or less, 2 patients in the group of more than 24 hours to 48 hours, and 3 patients in the group of more than 48 hours to 72 hours. The clopidogrel-aspirin group had a numerically lower risk of poor functional outcome at 90 days compared with the aspirin-alone group in patients with time to randomization of more than 48 hours to 72 hours (9.5% vs 10.8%; RR, 0.89 [95% CI, 0.69-1.14]) and more than 24 hours to 48 hours (8.8% vs 11.7%; RR, 0.74 [95% CI, 0.61-0.90]) but a numerically higher risk of poor functional outcome in those with time to randomization of 24 hours or less (14.8% vs 12.3%; RR, 1.01 [95% CI, 0.67-1.53]) (*P* = .94 for interaction) (Table 2). The results of other secondary efficacy outcomes are shown in Table 2, and there was no interaction effect between the antiplatelet treatment group and the time-to-randomization group.

**Table 2.** Efficacy and Safety Outcomes of Patients Among Different Times to Randomization

Outcome and time to randomization, h	Clopidogrel with aspirin (n = 3050)		Aspirin (n = 3050)		Unadjusted HR or RR (95% CI)^b^	*P* value	Adjusted HR or RR (95% CI)^b^^,^^c^	*P* value	*P* value for interaction
	Patients, No.	Events, No. (%)^a^	Patients, No.	Events, No. (%)^a^					
**Primary outcome**									
Stroke^d^									
≤24	401	46 (11.5)	382	51 (13.4)	0.84 (0.57-1.25)	.40	0.83 (0.55-1.25)	.38	.38
>24 to ≤48	1255	95 (7.6)	1297	116 (8.9)	0.84 (0.64-1.11)	.22	0.85 (0.65-1.12)	.25	
>48 to 72	1394	81 (5.8)	1371	112 (8.2)	0.70 (0.53-0.93)	.01	0.70 (0.53-0.94)	.02	
**Secondary outcome**									
Composite cardiovascular event^e^									
≤24	401	49 (12.2)	382	51 (13.4)	0.90 (0.61-1.33)	.58	0.87 (0.58-1.30)	.49	.29
>24 to ≤48	1255	97 (7.7)	1297	117 (9.0)	0.85 (0.65-1.12)	.25	0.86 (0.66-1.13)	.28	
>48 to 72	1394	83 (6.0)	1371	114 (8.3)	0.70 (0.53-0.93)	.01	0.71 (0.54-0.95)	.02	
Ischemic stroke									
≤24	401	42 (10.5)	382	50 (13.1)	0.78 (0.52-1.18)	.24	0.79 (0.52-1.21)	.28	.34
>24 to ≤48	1255	92 (7.3)	1297	114 (8.8)	0.83 (0.63-1.09)	.19	0.84 (0.64-1.11)	.22	
>48 to 72	1394	74 (5.3)	1371	110 (8.0)	0.65 (0.48-0.87)	.004	0.66 (0.49-0.89)	.007	
Hemorrhagic stroke									
≤24	401	4 (1.0)	382	1 (0.3)	3.84 (0.43-34.34)	.23	3.34 (0.23-48.14)	.38	.89
>24 to ≤48	1255	4 (0.3)	1297	2 (0.2)	2.08 (0.38-11.34)	.40	1.83 (0.32-10.44)	.50	
>48 to 72	1394	7 (0.5)	1371	2 (0.1)	3.44 (0.72-16.56)	.12	3.21 (0.64-16.15)	.16	
Myocardial infarction									
≤24	401	2 (0.5)	382	0	NA	NA	NA	NA	.40
>24 to ≤48	1255	1 (0.1)	1297	1 (0.1)	1.04 (0.07-16.56)	.98	NA	NA	
>48 to 72	1394	2 (0.1)	1371	1 (0.1)	1.97 (0.18-21.67)	.58	1.88 (0.16-22.02)	.62	
Vascular death									
≤24	401	5 (1.2)	382	2 (0.5)	2.39 (0.46-12.31)	.30	1.41 (0.22-9.15)	.72	.29
>24 to ≤48	1255	7 (0.6)	1297	4 (0.3)	1.81 (0.53-6.20)	.34	3.87 (0.79-18.90)	.09	
>48 to 72	1394	9 (0.6)	1371	9 (0.7)	0.98 (0.39-2.47)	.97	1.04 (0.39-2.79)	.94	
Poor functional outcome^f^									
≤24	400	59 (14.8)	381	47 (12.3)	1.20 (0.79-1.81)	.40	1.01 (0.67-1.53)	.95	.94
>24 to ≤48	1254	110 (8.8)	1296	151 (11.7)	0.75 (0.63-0.90)	.002	0.74 (0.61-0.90)	.002	
>48 to 72	1393	132 (9.5)	1369	148 (10.8)	0.88 (0.69-1.12)	.29	0.89 (0.69-1.14)	.35	
**Primary safety outcome**									
Moderate-to-severe bleeding^g^									
≤24	401	6 (1.5)	382	3 (0.8)	1.91 (0.48-7.64)	.36	1.57 (0.36-6.83)	.55	.92
>24 to ≤48	1255	9 (0.7)	1297	4 (0.3)	2.34 (0.72-7.59)	.16	2.25 (0.68-7.39)	.18	
>48 to 72	1394	12 (0.9)	1371	6 (0.4)	1.96 (0.74-5.23)	.18	2.00 (0.73-5.43)	.18	
**Secondary safety outcome**									
Death from any cause									
≤24	401	7 (1.7)	382	3 (0.8)	2.23 (0.58-8.62)	.25	1.77 (0.38-8.12)	.47	.80
>24 to ≤48	1255	15 (1.2)	1297	15 (1.2)	1.04 (0.51-2.12)	.92	1.30 (0.61-2.77)	.50	
>48 to 72	1394	15 (1.1)	1371	12 (0.9)	1.23 (0.58-2.62)	.60	1.31 (0.59-2.89)	.51	
Any bleeding^g^									
≤24	401	21 (5.2)	382	10 (2.6)	2.03 (0.96-4.31)	.07	1.66 (0.75-3.70)	.21	.51
>24 to ≤48	1255	44 (3.5)	1297	32 (2.5)	1.43 (0.91-2.25)	.13	1.40 (0.88-2.21)	.15	
>48 to 72	1394	29 (2.1)	1371	21 (1.5)	1.36 (0.77-2.38)	.29	1.30 (0.74-2.31)	.36	
Mild bleeding									
≤24	401	16 (4.0)	382	7 (1.8)	2.21 (0.91-5.37)	.08	1.71 (0.66-4.44)	.27	.25
>24 to ≤48	1255	37 (2.9)	1297	28 (2.2)	1.37 (0.84-2.24)	.21	1.33 (0.81-2.18)	.25	
>48 to 72	1394	17 (1.2)	1371	16 (1.2)	1.04 (0.53-2.06)	.91	1.00 (0.50-2.01)	>.99	
Intracranial hemorrhage									
≤24	401	4 (1.0)	382	2 (0.5)	1.91 (0.35-10.44)	.45	1.54 (0.22-10.74)	.66	.94
>24 to ≤48	1255	5 (0.4)	1297	2 (0.2)	2.60 (0.50-13.38)	.25	2.04 (0.38-10.94)	.41	
>48 to 72	1394	8 (0.6)	1371	4 (0.3)	1.96 (0.59-6.52)	.27	1.83 (0.53-6.31)	.34	

Abbreviations: HR, hazard ratio; NA, not applicable; RR, relative risk.

^a^ The percentage of events are based on the number of events divided by the number of patients.

^b^ The RRs are for poor functional outcome; the HRs are for other outcomes.

^c^ Adjusted for age, sex, medical history (previous ischemic stroke, diabetes, hypertension, or dyslipidemia), current smoking, systolic and diastolic blood pressure at admission, use of antiplatelet and lipid-lowering agents before qualifying event, qualifying event, symptomatic stenosis, National Institutes of Health Stroke Scale score, and statin treatment assignment.

^d^ Includes ischemic or hemorrhagic stroke.

^e^ Includes stroke, myocardial infarction, or death from cardiovascular causes.

^f^ Includes modified Rankin scale scores of 2 to 6 (range, 0 to 6, with higher scores indicating more disability and a score of 6 indicating death); data at 90 days were missing in 2 patients in the group of 24 hours or less and of more than 24 hours to 48 hours and in 3 patients in the group of more than 48 hours to 72 hours.

^g^ Bleeding events were defined according to the Global Utilization of Streptokinase and Tissue Plasminogen Activator for Occluded Coronary Arteries criteria.

### Safety Outcomes

**Figure 3.**
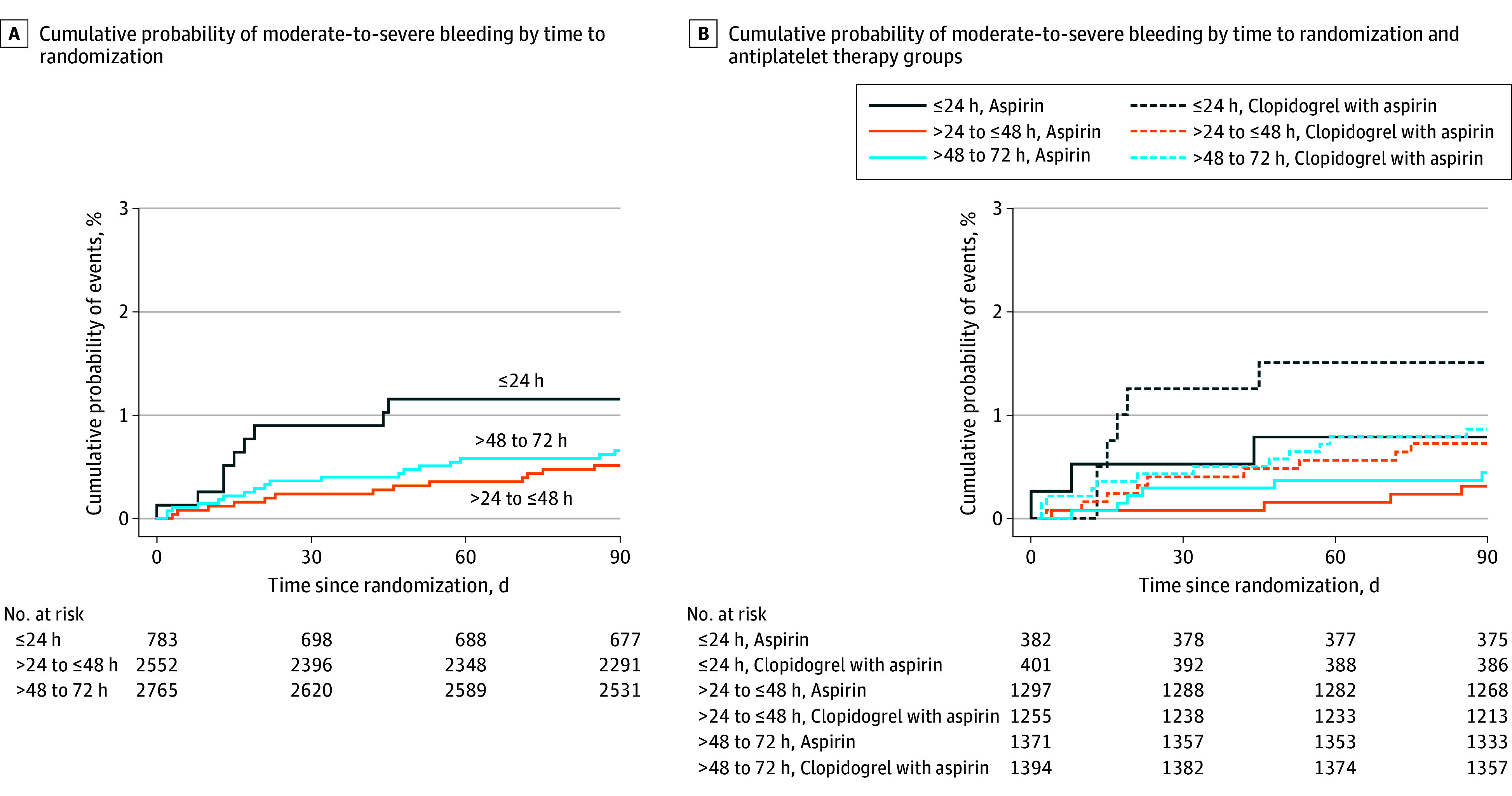
Kaplan-Meier Survival Curves of Cumulative Probability of Moderate-to-Severe Bleeding (Primary Safety Outcome) by Time to Randomization and Antiplatelet Therapy Groups

Compared with time to randomization of 24 hours or less, times to randomization of more than 24 hours to 48 hours (0.5% vs 1.2%; HR, 0.54 [95% CI, 0.22-1.30]; *P* = .16) and more than 48 hours to 72 hours (0.7% vs 1.2%; HR, 0.69 [95% CI, 0.29-1.60]; *P* = .38) was associated with lower risk of moderate-to-severe bleeding, the primary safety outcome (eTable 2 in Supplement 2 and Figure 3A). Among those with time to randomization of more than 48 to 72 hours, moderate-to-severe bleeding occurred in 12 patients (0.9%) in the clopidogrel-aspirin group and in 6 patients (0.4%) in the aspirin-alone group (HR, 2.00 [95% CI, 0.73-5.43]; risk difference, 0.39% [95% CI, 0.32%-0.42%]; number needed to harm, 256), while moderate-to-severe bleeding in those with time to randomization of more than 24 hours to 48 hours occurred in 9 patients (0.7%) in the clopidogrel-aspirin group and in 4 patients (0.3%) in the aspirin-alone group (HR, 2.25 [95% CI, 0.68-7.39]; risk difference, 0.44% [95% CI, 0.36%-0.52%]; number needed to harm, 227) and in those with time to randomization of 24 hours or less, occurred in 6 patients (1.5%) in the clopidogrel-aspirin group and in 3 patients (0.8%) in the aspirin-alone group (HR, 1.57 [95% CI, 0.36-6.83]; risk difference, 0.38% [95% CI, 0.04%-0.46%]; number needed to harm, 263) (*P* = .92 for interaction) (Table 2 and Figure 3B). Similar results were observed for secondary safety outcomes of death from any cause, any bleeding, mild bleeding, and intracranial hemorrhage. No significant interaction was observed between the time-to-randomization group and antiplatelet treatment assignment for other safety outcomes (*P* > .05 for all interactions) (Table 2).

## Discussion

In this exploratory subgroup analysis of the INSPIRES randomized clinical trial, we found that patients with mild ischemic stroke or high-risk TIA had a higher risk of new stroke within 90 days during earlier periods after symptom onset. The relative efficacy and safety of clopidogrel plus aspirin initiated within more than 24 hours to 48 hours and more than 48 hours to 72 hours were similar to that of clopidogrel and aspirin initiated within 24 hours or less. For patients with mild ischemic stroke or high-risk TIA, the risk of new stroke within 90 days may be reduced with DAPT with clopidogrel and aspirin by 15% when initiated within more than 24 hours to 48 hours and by 30% when initiated within more than 48 hours to 72 hours after symptom onset, with increases in the risk of moderate-to-severe bleeding similar across these time periods, although not statistically significant. Clopidogrel plus aspirin treatment appears beneficial and safe in all subgroups with time to randomization of 24 hours or less, more than 24 hours to 48 hours, and more than 48 hours to 72 hours.

Patients with acute ischemic stroke may have unstable atherosclerotic plaque and accelerated platelet aggregation in the early phase of the disease, resulting in a high risk of stroke recurrence and progression in the first few days.^11,12,17,18,19^ The main benefit of the early intensive antiplatelet therapy is to prevent this early risk of stroke recurrence. Previous trials focused on patients within 24 hours after symptom onset and demonstrated that DAPT was effective for minor stroke or high-risk TIA when initiated within 24 hours.^5,6,20^ In the present study, it is worth noting that the overall risk of new stroke was high among patients with time to randomization of 24 hours or less. New events occurred before enrollment were not collected and analyzed for patients with time to randomization of more than 24 hours. This supports that early intensive antiplatelet therapy should be emphasized for acute mild ischemic stroke and high-risk TIA. Although no statistically significant benefit of DAPT was observed in patients with time to randomization of 24 hours or less in this study, this finding may be related to the small number of patients enrolled in the study within 24 hours (n = 783), with low power (10%) to test the difference. Furthermore, after an alteration of the guidelines in 2019, those with an NIHSS score of 3 or less within 24 hours of ictus were excluded from the INSPIRES trial, so TIAs and those with lower NIHSS scores were excluded.^14^ The trial enrolled 783 patients within 24 hours of symptoms’ onset, including 211 patients (26.9%) with ischemic stroke and an NIHSS score of 4 to 5, which is different from the Clopidogrel in High-Risk Patients with Acute Non-Disabling Cerebrovascular Events^5^ and the Platelet-Oriented Inhibition in New TIA and Minor Ischemic Stroke^6^ trial populations (including patients with ischemic stroke and an NIHSS score of 3 or less). In addition, this study required the completion of cranial magnetic resonance and intracranial and extracranial vascular assessments to confirm compliance with the inclusion criteria.^14^ Completing these examinations within 24 hours posed practical implementation difficulties, excluding some of potential eligible patients. Although it was not significant, the point estimate of the HR for the primary outcome was 0.83 in patients with a time to randomization of 24 hours or less in the present study, indicating the potential benefit of DAPT considering the small sample size.

Extension of the treatment time window of DAPT may benefit more patients, since the current guidelines recommend a short time window (within 24 hours after symptom onset) of opportunity for secondary stroke prevention.^8,9^ Previous studies showed that the risk of new stroke was still high in the second and third days after symptom onset of minor stroke and TIA,^3,11,12^ indicating potential benefit from delayed DAPT for these patients. Previous meta-analysis^21^ and simulation study from the Platelet-Oriented Inhibition in New TIA and Minor Ischemic Stroke^22^ and the Acute Stroke or Transient Ischemic Attack Treated With Ticagrelor and Acetylsalicylic Acid for Prevention of Stroke and Death^23^ trials suggested that the benefit of DAPT in reducing major ischemic events may persist with a delay in initiation as late as 3 days after the onset of mild ischemic stroke or TIA. The recent Antiplatelet Therapy in Acute Mild-Moderate Ischemic Stroke trial showed that DAPT with clopidogrel and aspirin was superior to aspirin alone reducing early neurologic deterioration at 7 days in patients with acute mild to moderate ischemic stroke (NIHSS score of 4 to 10) presenting within 48 hours of symptom onset.^24^ The present subgroup analysis further provided additional evidence that DAPT with clopidogrel and aspirin had consistent benefit and risk between patients within more than 24 hours to 72 hours and those within 24 hours or less after symptom onset. Additionally, the absolute increase in risk of moderate-to-severe bleedings for clopidogrel-aspirin treatment was low: a 0.44% risk difference with a number needed to harm of 227 to produce 1 moderate-to-severe bleeding event within 90 days for patients with a time to randomization of more than 24 to 48 hours and a 0.39% risk difference with a number needed to harm of 256 for patients with a time to randomization of more than 48 hours to 72 hours.

### Limitations

There are several limitations in this study. First, this study is an exploratory subgroup analysis of a randomized clinical trial, and the findings should be considered as hypothesis generating, with low or modest power in each subgroup. Caution is warranted when interpreting the results of this study, especially regarding safety outcomes with small numbers of bleeding events. Second, the study did not involve ischemic stroke patients with an NIHSS score of more than 5 and nonatherosclerotic etiology. The extrapolation of the results of this study in these populations is limited. Third, patients who had already relapsed within 48 hours after ictus were excluded from those eligible to enroll in the time period of more than 48 hours to 72 hours of this study. Finally, the group enrolled within 24 hours or less excluded many TIAs and more minor strokes due to a protocol change required by shifting guidelines during the trial, so that time group may not be directly comparable with the others.

## Conclusions

In this exploratory subgroup analysis of the INSPIRES randomized clinical trial, patients with mild ischemic stroke or high-risk TIA had a higher risk of new stroke within 90 days in the earlier time periods after symptom onset, warranting early DAPT with clopidogrel and aspirin. This analysis suggests that these patients have a consistent benefit from receiving DAPT with clopidogrel and aspirin vs aspirin alone when initiated within more than 24 hours to 48 hours and more than 48 hours to 72 hours as that when initiated within 24 hours or less, with consistent increase in the risk of moderate-to-severe bleeding.

## Notes

Supplement 1.
Trial Protocol

Supplement 2. **eFigure.** Flow Diagram **eTable 1.** Baseline Characteristics of Patients With Different Times to Randomization **eTable 2.** Cumulative Probability of Stroke (Primary Efficacy Outcome) in Patients With Different Times to Randomization

Supplement 3.
Data Sharing Statement
