# Errors in Abstract and Methods

**DOI:** 10.1001/jamanetworkopen.2025.9310

**Published:** 2025-04-08

In the Original Investigation titled “Clopidogrel and Aspirin Initiated Between 24 to 72 Hours for Mild Ischemic Stroke: A Subgroup Analysis of the INSPIRES Randomized Clinical Trial,”^1^ published September 6, 2024, there were errors in the Abstract and the Methods sections. In the Interventions segment of the Abstract, the phrase given as “clopidogrel 300 mg loading dose on day 1, followed by 75 mg daily on days 2 to 90, and aspirin 100 to 300 mg on the first day and then 100 mg daily for days 2 to 90” should have been “clopidogrel 300 mg loading dose on day 1, followed by 75 mg daily on days 2 to 90, and aspirin 100 to 300 mg on the first day, 100 mg daily for days 2 to 21, and then a matching aspirin placebo for days 22 to 90.” In the Study Design section of the Methods, the phrase given as “clopidogrel 300 mg loading dose on day 1, followed by 75 mg daily on days 2 to 90, and aspirin 100 to 300 mg on the first day and then 100 mg daily for days 2 to 90” should have been “clopidogrel 300 mg loading dose on day 1, followed by 75 mg daily on days 2 to 90, and aspirin 100 to 300 mg on the first day, 100 mg daily for days 2 to 21, and then a matching aspirin placebo for days 22 to 90.” This article has been corrected.^1^
