# Supplementary material for: Clopidogrel and Aspirin Initiated Between 24 to 72 Hours for Mild Ischemic Stroke: A Subgroup Analysis of the INSPIRES Randomized Clinical Trial
Source: JAMA Netw Open. 2024 Sep 6;7(9):e2431938. doi: 10.1001/jamanetworkopen.2024.31938 (PMC11380102; doi:10.1001/jamanetworkopen.2024.31938)
Supplement: Supplement 2. — eFigure. Flow Diagram eTable 1. Baseline Characteristics of Patients With Different Times to Randomization eTable 2. Cumulative Probability of Stroke (Primary Efficacy Outcome) in Patients With Different Times to Randomization [file jamanetwopen-e2431938-s002.pdf]

Supplemental Online Content

Liu Y, Zhao J, Gao Y, et al. Efficacy and safety of clopidogrel and aspirin for mild ischemic stroke and transient ischemic attack: a subgroup analysis from the Intensive Medical Therapy for High-Risk Intracranial or Extracranial Atherosclerosis randomized clinical trial. *JAMA Netw Open*. 2024;7(9):e2431938. doi:10.1001/jamanetworkopen.2024.31938

eFigure. Flow Diagram
eTable 1. Baseline Characteristics of Patients With Different Times to Randomization
eTable 2. Cumulative Probability of Stroke (Primary Efficacy Outcome) in Patients With Different Times to Randomization

This supplemental material has been provided by the authors to give readers additional information about their work.

© 2024 Liu Y et al. *JAMA Network Open*.

eFigure. Flow diagram

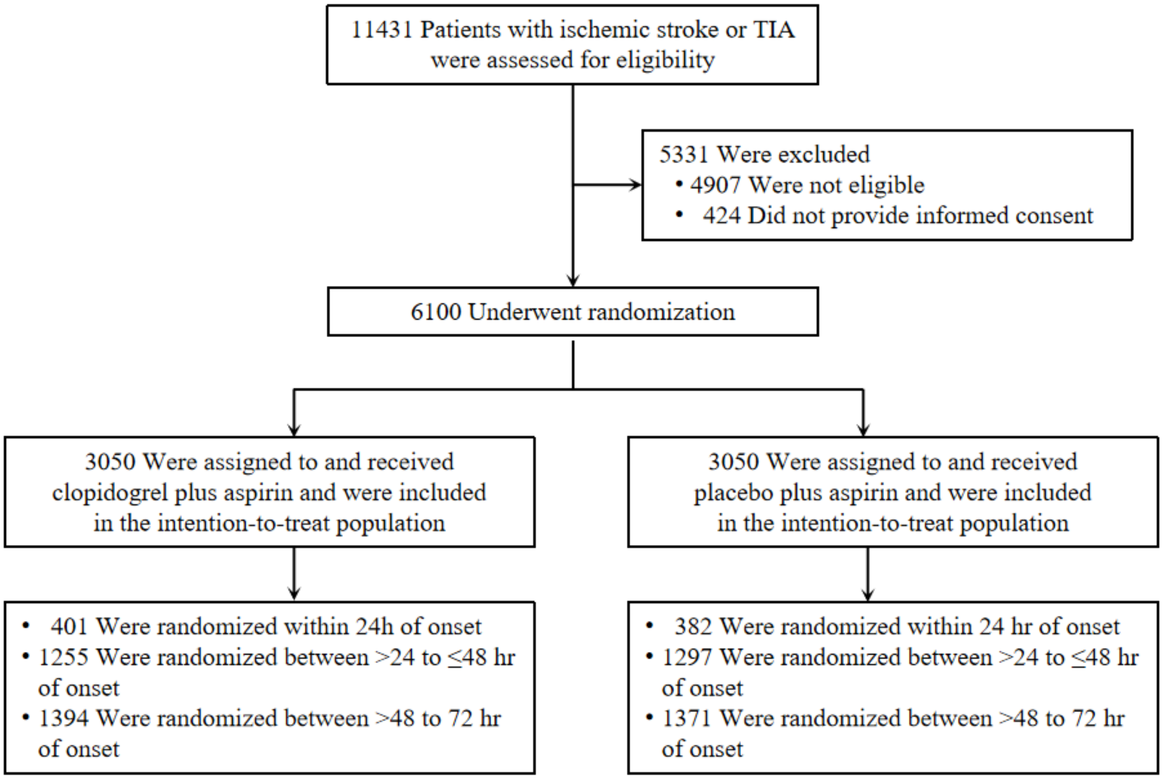

Abbreviations: TIA, transient ischemic attack.

© 2024 Liu Y et al. *JAMA Network Open*.

eTable 1. Baseline Characteristics of Patients With Different Times to Randomization

**Characteristics**	**Time to Randomization ≤24 hours (N= 783)**	**Time to Randomization >24 to ≤48 hours (N= 2552)**	**Time to Randomization >48 to 72 hours (N= 2765)**	**P value**
Age, mean (SD), y	65(57-71)	65(57-71)	65(57-71)	0.82
Female sex	268(34.2)	906(35.5)	1011(36.6)	0.44
Blood pressure, median (IQR), mmHg				
Systolic	149(135-162)	146(132-160)	145(132-160)	0.003
Diastolic	86(80-95)	85(78-94)	84(77-92)	<0.001
Medical history				
Hypertension	507(64.8)	1678(65.8)	1898(68.6)	0.03
Diabetes mellitus	196(25.0)	715(28.0)	747(27.0)	0.25
Dyslipidemia	35(4.5)	94(3.7)	97(3.5)	0.45
Previous ischemic stroke	236(30.1)	745(29.2)	828(29.9)	0.79
Current smoking	254(32.4)	767(30.1)	762(27.6)	0.01
Use of agents before qualifying event				
Aspirin	114(14.6)	338(13.2)	341(12.3)	0.23
Clopidogrel	8(1.0)	14(0.5)	21(0.8)	0.34
Lipid lowering agent	87(11.1)	242(9.5)	258(9.3)	0.31
Qualifying event				<0.001
TIA	142(18.1)	351(13.8)	308(11.1)	
Acute single infarction	148(18.9)	489(19.2)	537(19.4)	
Acute multiple infarctions	493(63.0)	1712(67.1)	1920(69.4)	
≥50% symptomatic stenosis				0.31
Yes	639(84.3)	2052(81.9)	2224 (82.2)	
No	119(15.7)	453(18.1)	481(17.8)	
NIHSS score in qualifying ischemic stroke [a]				<0.001
≤3	421(65.7)	1697(77.1)	1915(77.9)	
>3	220(34.3)	504(22.9)	542(22.1)	
$ABCD^2$ score in qualifying TIA [b]				0.11
4 or 5	117(82.4)	269(76.6)	255(82.8)	
>5	25(17.6)	82(23.4)	53(17.2)	
Statin treatment assignment				0.78
Immediate intensive statin	391(49.9)	1289(50.5)	1370(49.5)	
Delayed intensive statin	392(50.1)	1263(49.5)	1395(50.5)	

Abbreviations: IQR, interquartile range; TIA, transient ischemic attack; NIHSS, NIH Stroke Scale.

a. Scores on the NIHSS range from 0 to 42 for patients with ischemic stroke, with higher scores indicating more severe stroke.

b. The $ABCD^2$ score assesses the risk of stroke on the basis of age, blood pressure, clinical features, duration of TIA, and the presence or absence of diabetes mellitus in patients with transient ischemic attack. Scores range from 0 to 7, with higher scores indicating greater risk.

© 2024 Liu Y et al. *JAMA Network Open*.

eTable 2. Cumulative Probability of Stroke (Primary Efficacy Outcome) in Patients With Different Times to Randomization

Outcome	Time to Randomization	Patients	Events. No. (%)	Unadjusted HR/ RR (95% CI) [a]	P value	Adjusted HR/ RR (95% CI) [ab]	P value
**Primary outcome**							
Stroke (ischemic or hemorrhagic)	⩽24 hours	783	97(12.4)	Ref		Ref	
	>24 to ⩽48 hours	2552	211(8.3)	0.65(0.51-0.82)	<0.001	0.64(0.50-0.81)	<0.001
	>48 to 72 hours	2765	193(7.0)	0.54(0.42-0.69)	<0.001	0.52(0.41-0.67)	<0.001
**Secondary outcomes**							
Composite cardiovascular event (stroke, myocardial infarction, or death from cardiovascular causes)	≤24 hours	783	100(12.8)	Ref		Ref	
	>24 to ≤48 hours	2552	214 (8.4)	0.64(0.50-0.81)	<0.001	0.63(0.49-0.80)	<0.001
	>48 to 72 hours	2765	197(7.1)	0.54(0.42-0.68)	<0.001	0.52(0.40-0.66)	<0.001
Ischemic stroke	≤24 hours	783	92(11.8)	Ref		Ref	
	>24 to ≤48 hours	2552	206(8.1)	0.67(0.52-0.86)	0.001	0.65 (0.50-0.83)	<0.001
	>48 to 72 hours	2765	184(6.7)	0.55(0.43-0.70)	<0.001	0.52(0.40-0.67)	<0.001
Hemorrhagic stroke	≤24 hours	783	5(0.6)	Ref		Ref	
	>24 to ≤48 hours	2552	6(0.2)	0.37(0.11-1.20)	0.10	0.51(0.14-1.81)	0.30
	>48 to 72 hours	2765	9(0.3)	0.51(0.17-1.51)	0.22	0.67(0.20-2.24)	0.51
Myocardial infarction	≤24 hours	783	2(0.3)	Ref		Ref	
	>24 to ≤48 hours	2552	2(0.1)	0.31(0.04-2.18)	0.24	0.33(0.05-2.40)	0.27
	>48 to 72 hours	2765	3(0.1)	0.42(0.07-2.54)	0.35	0.56(0.09-3.46)	0.53
Vascular death	≤24 hours	783	7(0.9)	Ref		Ref	
	>24 to ≤48 hours	2552	11(0.4)	0.48(0.19-1.24)	0.13	0.49(0.17-1.38)	0.18
	>48 to 72 hours	2765	18(0.7)	0.73(0.30-1.74)	0.47	0.83(0.32-2.14)	0.70
Poor functional outcome (mRS 2-6) [c]	≤24 hours	783	106(13.6)	Ref		Ref	
	>24 to ≤48 hours	2552	261(10.2)	0.75(0.62-0.92)	0.005	0.82(0.67-1.02)	0.07
	>48 to 72 hours	2765	280(10.1)	0.75(0.59-0.95)	0.02	0.83(0.66-1.04)	0.11
**Primary safety outcome**							
Moderate-to-severe bleeding [d]	≤24 hours	783	9(1.2)	Ref		Ref	
	>24 to ≤48 hours	2552	13(0.5)	0.44(0.19-1.03)	0.06	0.54(0.22-1.30)	0.16
	>48 to 72 hours	2765	18(0.7)	0.56(0.25-1.25)	0.16	0.69(0.29-1.60)	0.38
**Secondary safety outcomes**							
Death from any cause	≤24 hours	783	10(1.3)	Ref		Ref	
	>24 to ≤48 hours	2552	30(1.2)	0.92(0.45-1.88)	0.82	1.05(0.49-2.22)	0.91
	>48 to 72 hours	2765	27(1.0)	0.76(0.37-1.58)	0.46	0.88(0.41-1.89)	0.74

© 2024 Liu Y et al. *JAMA Network Open.*

Any bleeding [d]	≤24 hours	783	31(4.0)	Ref		Ref	
	>24 to ≤48 hours	2552	76(3.0)	0.75(0.49-1.14)	0.18	0.94(0.61-1.46)	0.78
	>48 to 72 hours	2765	50(1.8)	0.45(0.29-0.71)	<0.001	0.55(0.34-0.89)	0.02
Mild bleeding	≤24 hours	783	23(2.9)	Ref		Ref	
	>24 to ≤48 hours	2552	65(2.6)	0.87(0.54-1.40)	0.56	1.10(0.66-1.82)	0.72
	>48 to 72 hours	2765	33(1.2)	0.40(0.24-0.69)	<0.001	0.50(0.28-0.87)	0.02
Intracranial hemorrhage	≤24 hours	783	6(0.8)	Ref		Ref	
	>24 to ≤48 hours	2552	7(0.3)	0.36(0.12-1.06)	0.06	0.47(0.15-1.49)	0.18
	>48 to 72 hours	2765	12(0.4)	0.56(0.21-1.50)	0.25	0.75(0.26-2.18)	0.60

Abbreviations: HR, hazard ratio; RR, relative risk; CI, confidence interval; mRS, modified Rankin scale; NA, not applicable.

[a] The relative risks are shown for poor functional outcome. Hazard ratios are shown for other outcomes.

[b] adjusted for age, sex, medical history (previous ischemic stroke, diabetes mellitus, hypertension, dyslipidemia), current smoking, systolic and diastolic blood pressure at admission, use of antiplatelet and lipid lowering agents before qualifying event, qualifying event, symptomatic stenosis, NIHSS score, antiplatelet and statin treatment assignment.

[c] The mRS score data at 90 days were missing in 2 patients in the ≤24 hours group, 2 patients in the >24 to ≤48 hours group and 3 patients in the >48 to 72 hours group.

[d] Bleeding events were defined according to the Global Utilization of Streptokinase and Tissue Plasminogen Activator for Occluded Coronary Arteries criteria.

© 2024 Liu Y et al. *JAMA Network Open*.
