# Supplementary material for: Clopidogrel and Aspirin Initiated Between 24 to 72 Hours for Mild Ischemic Stroke: A Subgroup Analysis of the INSPIRES Randomized Clinical Trial
Source: JAMA Netw Open. 2024 Sep 6;7(9):e2431938. doi: 10.1001/jamanetworkopen.2024.31938 (PMC11380102; doi:10.1001/jamanetworkopen.2024.31938)
Supplement: Supplement 3. — Data Sharing Statement [file jamanetwopen-e2431938-s003.pdf]

Data Sharing Statement

Liu. Clopidogrel and Aspirin Initiated Between 24 to 72 Hours for Mild Ischemic Stroke. *JAMA Netw Open*. Published September 06, 2024. doi:10.1001/jamanetworkopen.2024.31938

Data
Data available: Yes
Data types: Deidentified participant data
How to access data: Anonymized data are available to researchers on request for reproducing the results or replicating the procedures by contacting the corresponding author(yilong528@aliyun.com).
When available: With publication

Supporting Documents
Document types: None

Additional Information
Who can access the data: Researchers whose proposed use of the data has been approved
Types of analyses: Researchers on request for reproducing the results or replicating the procedures
Mechanisms of data availability: After approval of a proposal
